# CDC’s National Asthma Control Program: Public Health Actions to Reduce the Burden of Asthma

**DOI:** 10.5888/pcd21.240344

**Published:** 2024-09-19

**Authors:** Maria C. Mirabelli, Hailay Teklehaimanot, Tyra Bryant-Stephens

**Affiliations:** 1 Asthma and Air Quality Branch, Division of Environmental Health Science and Practice, National Center for Environmental Health, Centers for Disease Control and Prevention, Atlanta, Georgia; 2 Department of Pediatrics, Children’s Hospital of Philadelphia, Philadelphia, Pennsylvania

Across the US, public health and clinical institutions work to meet the health care needs of children and adults with asthma (1–3). This work ranges from basic research aimed at discovering the causes, predictors, and environmental triggers of asthma (2,3) to translational activities focused on improving access to effective medications to improve asthma control and implementation of evidence-based interventions in diverse settings to improve asthma outcomes among children and adults with asthma (4–9). Despite these efforts, asthma continues to be a common chronic health condition in the US, especially among school-aged children (10). Although there is no cure for asthma, remarkably effective treatments exist that can decrease asthma exacerbations and improve quality of life among those living with asthma (11).

## CDC’s National Asthma Control Program

The Centers for Disease Control and Prevention’s (CDC’s) National Asthma Control Program (NACP) is the primary source of funding for state, tribal, local, and territorial agencies to establish and maintain asthma public health programs. The NACP supports these agencies to reduce the numbers of deaths, hospitalizations, emergency department visits, missed school days, missed workdays, and activity limitations due to asthma (1). CDC initiated the NACP in 1999 and since then has supported the planning and development of state, tribal, local, and territorial asthma control programs to conduct asthma surveillance, develop asthma interventions, evaluate the effectiveness of those interventions, and form partnerships to implement them in the communities that need them most (1,12).

This collection, *Public Health Actions to Reduce the Burden of Asthma*, consists of 9 peer-reviewed articles that highlight the history of CDC-funded asthma control programs in the US, the burden of asthma among those most affected, and examples of the development, implementation, and evaluation of asthma-related interventions to improve asthma control. These articles provide readers with examples of the activities asthma control programs conduct to describe and reduce the negative impact of asthma on the lives of people with asthma and their families.

To introduce readers to CDC’s approach to improving asthma control, Etheredge et al describe the history of the NACP and the initial use of asthma surveillance to understand and raise awareness about the burden of asthma in the US (12). At the onset of the NACP, funded asthma control programs established surveillance of asthma-related emergency department visits and used the surveillance data to better understand the burden of asthma in the communities they served (12). Over time, the programs expanded to include the use of evidence-based interventions to improve asthma control, the development of partnerships with professional and community organizations, and the evaluation of the program-led surveillance, partnerships, and interventions (12). Most recently, the NACP has focused on building sustainable partnerships to expand the implementation of evidence-based strategies to address the persistent disparities in the prevalence and severity of asthma and its related outcomes (12).

## Prevalence and Burden of Asthma

Several articles in this collection combine traditional and novel analytic methods to describe the US prevalence of asthma, asthma attacks, asthma-related health care use, and risk factors for asthma exacerbations (13–17). Surveillance data reported by Pate and Zahran provide insight into not only the prevalence of current asthma, asthma-related health care use, and asthma mortality but also trends in these outcomes over the past decade (13). In these data, the observed decrease over time in the prevalence of asthma among children, coupled with a decrease in the occurrence of reported asthma attacks, asthma emergency department visits, asthma hospitalizations, and asthma mortality among children suggests that US children might be experiencing improved asthma control; however, the differences reported across race and ethnicity and other characteristics indicate that asthma health disparities persist (13). A decline over time in US pediatric asthma hospitalizations is also highlighted by Binney et al, who show that while the similarity of the rates of decline across all racial and ethnic groups indicates progress in reducing the burden of asthma overall, disparities by race and ethnicity continue (14). Wang and Nurmagambetov extend our understanding of the burden of asthma among US children by reporting on the additional annual medical expenditures for children with treated asthma compared with children without treated asthma (15). Again, differences by race and ethnicity show the complexities affecting disparities in asthma control; although medical expenditures were lower for non-Hispanic Black children than for non-Hispanic White children with treated asthma, the findings raise important questions about the differences in asthma-related health care use and the financial burden experienced across racial and ethnic groups. Together, these 3 articles can guide public health and health care professionals in identifying patient groups most in need of effective and low-cost approaches to reduce the burden of asthma.

Asthma disparities also occur across geographic areas. Skochko et al applied an emerging hot spot analysis approach to identify high-burden areas in New York before and during the COVID-19 pandemic (16). This approach identified local variations in asthma emergency department visits that indicate geographic areas in which local, evidence-based asthma interventions might be especially effective.

Authors of these 4 articles each point out that some people or populations continue to experience a disproportionately greater burden of asthma. Conversely, Jaffee et al describe differences in influenza and COVID-19 vaccination rates among US adults with asthma across demographic, geographic, and demographic characteristics (17). Despite high levels of vaccination overall, variations in vaccination rates among adults with asthma indicate opportunities for education-based interventions about the benefits of vaccinations for adults with asthma, especially younger adults and adults in rural areas. Jaffee et al provide us with important information about differences in health behaviors that can affect the risk of exacerbations among people with asthma (17).

## Asthma Control Activities and Evaluation

The NACP supports the development and implementation of evidence-based interventions to address the burden of asthma in the US, including the asthma disparities identified through surveillance and other assessments. Articles in this collection provide examples of how state asthma control programs and partners implement and evaluate the interventions designed to improve asthma control (18,19). One such evaluation is described by Wing et al, who report on the use of a toolkit, *Supporting Students with Asthma at School: Standards of Care,* to prepare school nurses with information about asthma, asthma management, applicable laws, and other aspects of supporting students with asthma (18). Their evaluation of the effectiveness of the toolkit identified important barriers to its use, such as time and parent engagement, as well as notable successes in training and education, the use of asthma action plans, and advocacy for medication self-carry policies. Mahin et al report on the use of a community health worker–led asthma home visiting program and the projected cost savings of its expansion to improve asthma outcomes among pediatric Medicaid patients with uncontrolled asthma (19). Their findings projected that expansion of the community health worker–led home visiting program would result in a $566.58 per-patient reduction in the 2019 costs associated with asthma emergency department visits and hospitalizations. These 2 articles provide compelling examples of how programs that support school nursing staff and community health worker home visits for asthma can improve support and lower costs, respectively, for children with asthma.

Evidence of the impact of interventions such as those described by Wing et al (18) and Mahin et al (19) often comes from evaluation of the interventions, as well as from evaluation of the surveillance systems and partnerships that support the development and implementation of such interventions. Indeed, the NACP recognizes that evaluation is an important tool for learning how to improve programs (20). In this collection, Dunklin et al (20) describe the history of the development of evaluation methods that have led the NACP and its funding recipients and partners to identify some of their most effective interventions and partnerships. The authors describe the development and use of evaluation tools and provide an inventory of tools developed by the NACP (20). The evaluation tools described are still the primary resources used today to assess and improve specific components of asthma control programs, leading to more cost-effective and engaging programs (20).

## Future Direction

CDC’s NACP will continue to focus on improving the lives of people and communities most affected by asthma. To increase the number of people with asthma whose health is improved because of the asthma control programs where they live and to maximize the impact of these programs, the NACP is prepared to pursue 3 approaches in the coming years:

1. **Strategic investment of resources** on cost-efficient, scalable, and sustainable public health interventions, with a focus on implementing interventions in communities most affected by asthma
2. **Strong partnerships** across multiple sectors to more effectively develop and implement evidence-based asthma interventions
3. **Environmental health guidance** that can be used across the US to reduce indoor, outdoor, and occupational asthma triggers

To support these approaches, the next iteration of CDC’s NACP, Advancing Health Equity in Asthma Control Through EXHALE Strategies, will focus on funding programs to address the environmental, social, and systematic drivers of existing disparities in asthma (21). The intention is that asthma control programs will accomplish this by strengthening the relationships with their partners to implement EXHALE strategies (22). EXHALE strategies are described in CDC’s EXHALE Technical Package (22) and include 6 approaches proven to reduce asthma-related emergency department visits, hospitalizations, and health care costs. The strategies are: E = Education on asthma self-management; X = X-tinguish smoking and exposure to secondhand smoke among people with asthma; H = Home visits for trigger reduction and asthma self-management education; A = Achievement of guidelines-based medical management; L = Linkages and coordination of care across settings; E = Environmental policies or best practices to reduce asthma triggers from indoor, outdoor, and occupational sources (22). By supporting programs to implement these strategies during its next funding cycle, the NACP will strengthen sustainable and effective leadership, program management, partnerships, surveillance, health communication, and program evaluation. Successful implementation of these strategies will contribute to the reduction in asthma-related emergency department visits, hospitalizations, and health care costs in the populations that need them most.

## Conclusions

This collection shares articles highlighting successes of CDC’s NACP over the past 25 years. The insights and findings identified by CDC, funded asthma control programs, and other partners offer examples of evidence-based asthma interventions that can be built on moving forward. Reducing asthma health disparities by improving asthma control among people most affected by asthma should improve the lives and health of everyone affected by asthma. Fewer emergency department visits, hospital stays, school and workdays missed, medical expenses, and deaths due to asthma improve the lives of people with asthma and those who care for them.
